# Isolation and characterization of plant growth-promoting rhizobacteria from wheat rhizosphere and their effect on plant growth promotion

**DOI:** 10.3389/fmicb.2015.00198

**Published:** 2015-03-17

**Authors:** Afshan Majeed, M. Kaleem Abbasi, Sohail Hameed, Asma Imran, Nasir Rahim

**Affiliations:** ^1^Department of Soil and Environmental Sciences, The University of PoonchRawalakot, Azad Jammu and Kashmir, Pakistan; ^2^National Institute for Biotechnology and Genetic EngineeringFaisalabad, Pakistan

**Keywords:** biofertilizers, diversity, plant growth-promoting rhizobacteria, 16S rRNA, wheat

## Abstract

The present study was conducted to characterize the native plant growth promoting (PGP) bacteria from wheat rhizosphere and root-endosphere in the Himalayan region of Rawalakot, Azad Jammu and Kashmir (AJK), Pakistan. Nine bacterial isolates were purified, screened *in vitro* for PGP characteristics and evaluated for their beneficial effects on the early growth of wheat (*Triticum aestivum* L.). Among nine bacterial isolates, seven were able to produce indole-3- acetic acid in tryptophan-supplemented medium; seven were nitrogen fixer, and four were able to solubilize inorganic phosphate *in vitro*. Four different morphotypes were genotypically identified based on IGS-RFLP fingerprinting and representative of each morphotype was identified by 16S rRNA gene sequencing analysis except Gram-positive putative *Bacillus* sp. Based on 16S rRNA gene sequence analysis, bacterial isolates AJK-3 and AJK-9 showing multiple PGP-traits were identified as *Stenotrophomonas* spp. while AJK-7 showed equal homologies to *Acetobacter pasteurianus* and *Stenotrophomonas* specie. Plant inoculation studies indicated that these Plant growth-promoting rhizobacteria (PGPR) strains provided a significant increase in shoot and root length, and shoot and root biomass. A significant increase in shoot N contents (up to 76%) and root N contents (up to 32%) was observed over the un-inoculated control. The study indicates the potential of these PGPR for inoculums production or biofertilizers for enhancing growth and nutrient content of wheat and other crops under field conditions. The study is the first report of wheat associated bacterial diversity in the Himalayan region of Rawalakot, AJK.

## Introduction

Chemical fertilizers are generally used to supply essential nutrients to the soil–plant system throughout the world. However, the prices, availability, and the environmental concerns of chemical fertilizers especially the N fertilizers are real issues of today’s agriculture. Application of chemical fertilizers in slopping landscapes under high annual rainfall normally exist in the mountain ecosystem of the Hindu Kush Himalayan (HKH) region may not be effective because of surface runoff and leaching. Therefore, there is an urgent need to find alternative strategies that can ensure competitive crop yields, provide environmental safety, and protection while maintain long term ecological balance in agro-ecosystem. Use of microbial inoculants or plant growth-promoting rhizobateria (PGPR) for the enhancement of sustainable agricultural production is becoming a more widely accepted practice in intensive agriculture in many parts of the Plant growth-promoting rhizobacteria are free-living soil bacteria that aggressively colonize the rhizosphere/plant roots, and enhance the growth, and yield of plants when applied to seed or crops (Kumar et al., 2014). The plant growth promoting (PGP) effect of the PGPR is mostly explained by the release of metabolites directly stimulating growth. Several mechanisms have been postulated to explain how PGPR benefit the host plant. These include: (a) the ability to produce plant growth regulators or phytohormones such as indole acetic acid (IAA), cytokinins, and gibberellins (Glick, 1995; Marques et al., 2010); (b) enhancing asymbiotic N_2_ fixation (Sahin et al., 2004; Khan, 2005); (c) solubilizing inorganic phosphate and mineralization of organic phosphate and/ or other nutrients (Glick, 1995; Jeon et al., 2003); (d) antagonistic effect against phytopathogenic microorganisms by production of siderophores, the synthesis of antibiotics, enzymes, and/or fungicidal compounds, and competition with detrimental microorganisms (Dey et al., 2004; Lucy et al., 2004).

Interest in the beneficial rhizobacteria associated with cereals has increased recently and several studies clearly demonstrated the positive and beneficial effects of PGPR on growth and yield of different crops especially wheat at different environment under variable ecological conditions (Ozturk et al., 2003; Marques et al., 2010; Mehnaz et al., 2010; Zhang et al., 2012). Inoculation with *Pseudomonas fluorescens* showed a significant increase in root weight 19–43%, number of tillers per plant 10–21%, grain yield 15–43%, and straw yield 22–39% of wheat compared to un-inoculated plants (Shaharoona et al., 2008). Moreover, inoculation with PGPR strain *Azotobacter* saved 25–30 kg N ha^-1^ chemical fertilizer (Narula et al., 2005). More recently, Kumar et al. (2014) conducted experiments on wheat under pot and field condition to examine the effect of PGPRs on the growth and yield of wheat and found that triple combination of strains *B. megaterium*, *A. chlorophenolicus,* and *Enterobacter* significantly increased 17.5, 79.8, 78.6, and 26.7% plant height, grain yield, straw yield, and test weight under pot condition and also 29.4, 27.5, 29.5, and 17.6% under field condition, respectively.

Knowledge of the native bacterial population, their characterization, and identification is required for understanding the distribution and diversity of indigenous bacteria in the rhizosphere of specific crops (Keating et al., 1995; Chahboune et al., 2011). With increasing awareness about the-chemical-fertilizers-based agricultural practices, it is important to search for region-specific microbial strains which can be used as a growth promoting/enhancing inoculum to achieve desired crop production (Deepa et al., 2010). Recently, the bacterial diversity in the forest soil of Kashmir, India was investigated and reported (Ahmad et al., 2009) but no data is available regarding the rhizosphere microbiome of wheat native to this area. Wheat being a staple food has special importance in the economy of the country.

Keeping in mid the study was planned to isolate the native strains from rhizosphere and endo-rhizosphere of wheat grown on different soils of Rawlakot, AJK. These bacteria were characterized and screened *in vitro* for PGP potentials and representative isolates were identified by 16S rRNA sequence analysis. Furthermore, the PGP potential was evaluated *in vivo* under axenic conditions and effect on the growth, and N contents of wheat at early growth stage was investigated.

## Materials and Methods

### The Study Site

The study site is located in an experimental farm of the University of the Poonch Rawalakot, Azad Jammu and Kashmir (AJK), Pakistan at the Faculty of Agriculture Rawalakot. Rawalakot is located at latitude 33^∘^51^′^32.18^′′^N, longitude 73^∘^ 45^′^34.93^′′^E, and an elevation of 1638 m above the sea level in the north–east of Pakistan under the foothills of great Himalayas. The topography is mainly hilly and mountainous with valleys and stretches of plains. The area is characterized by a temperate sub-humid climate with annual average rainfall ranging from about 500-2000 mm, most of which is irregular and falls with intense storms during monsoon and winter. The monthly mean temperature ranges from a minimum of 0^∘^C to a maximum of 22^∘^C accompanied by a severe cold and snow fall in winter. The soil used in the study (0–15 cm) was silt loam in texture (Organic carbon 9.5 g kg^-1^, total N 1.02 g kg^-1^, available P 2.5 mg kg^-1^, available K 54 mg kg^-1^ and pH 6.7).

### Sample Collection and Isolation of Bacteria

Wheat (*Triticum aestivum* L.) variety Inqlab–91, plant samples were collected from Research farm fields of Faculty of Agriculture Rawalakot along with bulk rhizospheric soil. Samples were placed individually in plastic bags and brought to National Institute of Biotechnology and Genetic Engineering (NIBGE), Faisalabad for isolation of bacteria. Rhizospheric bacteria were isolated from 1 g soil tightly adhering to the root by serial dilution plating on Luria–Bertani (LB) agar plates as described (Somasegaran and Hoben, 1994). Endophytic bacteria were isolated by serial dilution plating of sterilized crushed root samples on LB agar plates as described (Hameed et al., 2004). The plates were incubated at 28 ± 2^∘^C till the appearance of bacterial colonies. Individual colonies were picked and streaked on LB plates for further purification.

### Biochemical Characterization

Colony morphology, size, color, shape, gum production, and growth pattern were recorded after 24 h of growth on LB agar plates at 28 ± 2^∘^C as described by Somasegaran and Hoben (1994). Cell size and motility was observed by light microscopy. Acid/alkali production was tested on LB agar plates containing 0.025% (w/v) bromothymol blue as pH indicator. The Gram reaction was performed as described by Vincent and Humphrey (1970). Amino-peptidase and cytochrome oxidase tests were performed by using commercially available strips (Merck, Darmstadt, Germany). Catalase production was checked by placing a drop of H_2_O_2_ onto the bacterial colony on a glass slide.

### Molecular Characterization

Total genomic DNA of bacterial strains was extracted by alkaline lysis method (Maniatis et al., 1982) with slight modifications. DNA concentration was determined by DNA fluorometer (DYNA Quant^TM^ 200). On the basis of cell and colony morphology, nine different morphotypes were identified from rhizosphere and endosphere of wheat. These morphotypes were subjected to restriction fragment length polymorphism of IGS to distinguish among these genotypes. 16S rRNA–23S rRNA intergenic spacer region was amplified as described (Laguerre et al., 1996) using forward primer FGPS-1490 (5-TGGGGCTGGATCACCTCCTT -3) and reverse primer FGPS-132 (5- CCGGGTTTCCCCATTCGG-3). The PCR-product was restricted using BamHI and HindIII bacterial isolates were clustered. A representative of each IGS-RFLP type was identified by analysis of 16S rRNA gene sequence. The complete 1.5-kb 16S rRNA region was amplified using the universal bacterial 16S rRNA primers P1 a forward primer (P1 = 5^′^- cgggatccAGAGTTTGATCCTGGTCAGAACGAACGCT -3^′^) and P6; a reverse primer (P6 = 5^′^- cgggatccTACGGCTACCTTGTTACGACTTCACCCC -3^′^) which correspond to *Escherichia coli* positions 8–37 and 1479–1506, respectively, and amplifies 1500 bp full gene fragment (Tan et al., 1997).

The PCR amplification of the target sequence was done as described (Imran et al., 2010). Amplified PCR products of 16S ribosomal gene were confirmed on 1% agarose gel, purified using QIAquick PCR purification kit (QIAGEN) and cloned into *E. coli* TOP10 (Invitrogen). Plasmids DNA was isolated using Rapid plasmid mini Prep kit (Marligen Bioscience) and restricted with *EcoRI* and *BamHI* for the confirmation of transformed amplicons. Cloned PCR products were sequenced commercially by Macrogen (Korea).

The obtained gene sequences were compared with others in the Gen Bank databases using the NCBI BLAST at http://www.ncbi.n1m.nih.gov/blast/Blast.cgi. Sequences were submitted to NCBI GenBank data base and accession numbers were obtained.

### Bioassays for Plant Growth Promoting Traits

#### Solubilization of Insoluble Phosphate and Zinc

Each bacterial culture was spot inoculated in the center of agar plates containing tricalcium phosphate as insoluble phosphate source (Pikovskaya, 1948) and onto LGI medium (Cavalcante and Döbereiner, 1988) supplemented with 0.1% zinc oxide and zinc sulfate. The plates were incubated at 28 ± 2^∘^C for 7–10 days and observed for the formation of halo zone around the colonies. P-solubilization was quantified by Phospho-molybdate blue color method using spectrophotometer (λ = 882) as described by Murphy and Riley (1962). The experiment was repeated twice with three replicates each and mean was calculated.

#### Production of Indole-3-Acetic Acid

Bacterial cultures were grown in Okon et al.’s (1977) malate medium supplemented with tryptophan (100 mg/L) as the precursor of IAA and compared to those grown without the addition of tryptophan precursor. IAA production was determined using colorimetric methods (Gordon and Weber, 1951) and quantified on HPLC using ethyl acetate oxidation method (Tien et al., 1979). The experiment was repeated twice with three replicates each and mean was calculated.

#### Nitrogen Fixation

Nitrogenase activity was detected by acetylene reduction/ethylene production assay as described earlier (Mirza et al., 2001). Pure bacterial colonies were inoculated into NFM (Nitrogen Free Malate) semisolid medium vials and incubated at 28 ± 2^∘^C for 48 h. Acetylene (10% v/v) was injected to the vials, incubated at 28 ± 2^∘^C for 16 h and 100 μL of gas samples from the vials were analyzed on a gas chromatograph (Thermoquest, Trace G.C, Model K, Rodono Milan, Italy) equipped with a Porapak Q column and a H_2_-flame ionization detector (FID) using conditions described in Hardy et al. (1973). The experiment was repeated twice with three replicates each and mean was calculated.

### Plant Inoculation and Root Colonization Assays

Effect of bacterial inoculation on plant growth was examined on wheat variety Inqalab–91 in a growth room experiment. The *Azospirillum brasilense* strain Er-20 (Mirza et al., 2000) was used as positive control while un-inoculated plants served as negative control. Bacterial cultures were grown in 50 mL falcon tubes filled with 25 mL LB broth and were kept on shaker at 200 rpm for 16 h. Seeds were germinated on water agar plates and transferred aseptically to plant growth pouches (Weaver and Frederick, 1982) after 4 days of seedling emergence. Before transferring the germinated seeds to the pouches (autoclaved), 20 mL of 1/2 strength Hoagland’s nutrient solution was poured in the pouches. The un-inoculated pouches were watered with full strength Hoagland (with N, P) while inoculated pouches received full strength Hoagland without N and P along-with 1 g inorganic tri-calcium phosphate as sole P-source. After 2 days of seedling transplant, 1 mL inoculum (cells suspended in 0.85% saline; OD = 0.45) was applied at the base of each seedling. The un-inoculated pouches were supplied with same amount of sterile saline. Two seedlings were maintained per growth pouch and placed in growth chamber at 20–22^∘^C with a day length of 12 h and relative humidity was set at 70%. The experiment was set up in randomized complete design (CRD) using 10 replicate pouches per treatment. Plants were harvested 60 days after transplantation and data was recorded for shoot and root length, shoot and root dry weight, and shoot and root N contents. Total nitrogen contents of the plants were determined by the Kjeldahl method (Keeney and Nelson, 1982).

Root colonization potential of inoculated bacteria was determined at every 15 day using serial dilution plating technique on LB agar and number of viable cells was estimated as colony forming units (CFU) as described in Somasegaran and Hoben (1994) on LB agar plates.

### Statistical Analysis

The data were subjected to analysis of variance using statistical program xxx (MSTATC, 1990). The differences among various treatment means were compared using the least significant differences test (LSD) at 5% (*P* ≤ 0.05) probability level (Steel and Torrie, 1980).

## Results

### Culturable Bacteria in the Rhizosphere and Endosphere of Wheat

Bacteria were obtained both from rhizospheric portion as well as root interior of wheat. Six bacteria were obtained from wheat rhizosphere soil while three from root interior (**Table 1**). The bacteria showed gummy, white-to milky white colonies with variable sizes and margins (**Table 1**) on LB agar plates. The cells were mostly motile, rod shaped showing Gram-negative reaction except AJK-4 and AJK-8 both of which were tentatively identified as *Bacillus* sp. based on their colony and cell morphologies and Gram reaction.

**Table 1:** Morphological characteristics of bacterial isolates from the wheat rhizosphere in the mountain region of Rawalakot, Azad Jammu and Kashmir (AJK), Pakistan.

Strains	Isolated from	Colony size and shape	Colony color	Acid/alkali production on BTB	Cell motility	Cell shape	Gram reaction
AJK-1	RHS	Small, Wavy	White	Neutral	Highly motile	Small rods	-
AJK-2	RHS	Small, Round	Milky white	Neutral	Motile	Medium rods	-
AJK-3	RHS	Large, Round	Dark Yellow	Acidic	Highly motile	Oval	-
AJK-4	RHS	Medium, Wavy	Off-white	Neutral	Slowly motile	oval	+
AJK-5	RHS	Small, Round	Off-white	Acidic	Slowly motile	Medium rods	-
AJK-6	RHS	Large, Round	Dark yellow	Acidic	Highly motile	Small rods	-
AJK-7	RI	Medium, Round	Milky white	Neutral	Highly motile	Thin rods	-
AJK-8	RI	Medium, Round	Milky white	Neutral	Highly motile	Small rods	+
AJK-9	RI	Small, Round	Milky white	Neutral	Highly motile	Medium rods	-

RHS, Rhizosphere soil; RI, Root interior.

### Identification of Bacteria

Nine wheat rhizo/endophytic bacteria yielded four IGS patterns. Among these four patterns, one comprising of *Bacillus* sp. was not sequenced but from other three types, one strain each was sequenced based on 16S rRNA gene. The PGPR isolate AJK-3 was identified as *Stenotrophomonas rhizophila* strain having 99% similarity with the reported gene sequence. AJK-7 showed 98% similarity with *Acetobactor pasteurianus*. However, isolate AJK-9 95% similarity with *Stenotrophomonas* specie. The new isolate, AJK-9 had many of its beneficial characteristics resembling *Stenotrophomonas* strain AJK-3, hence may be considered as a *Stenotrophomonas* species. Nucleotide accession number for strain AJK-9**,** AJK**-**7, and AJK-3 are GQ130134, GQ130133, and GQ130132, respectively.

### Plant Growth Promoting Potential

*In vitro* plant growth promotion traits of the rhzio/endophytic bacteria are described in **Table 2**. Seven out of nine bacterial isolates (except isolates AJK-2, AJK-5) were able to produce IAA with a range of 0.27–77.98 μg ml^-1^ in the presence of IAA precursor tryptophan. *Stenotrophomonas* sp. strain AJK-9 produced maximum IAA (77.98 μg ml^-1^) followed by *S. rhizophila* strain AJK-3. The isolates AJK-1, AJK-2, AJK-3, and AJK-9 produced IAA even without IAA precursor tryptophan.

**Table 2:** Biochemical and molecular analysis of rhizosphere and endophytic bacteria isolated from wheat rhizosphere from Rawlakot, AJK.

Isolates^∗^	Biochemical analysis						Molecular analysis
	Catalase activity	Cytochrome oxidase	P-solubilization (mg/mL)	IAA (μg/mL) (with Tryptophan)	IAA (μg/mL) (without Tryptophan)	ARA (μmole C_2_H_4_mg protein/h)	Identification based on IGS type/biochemical/16S rRNA gene sequencing
AJK-1	+	+	17	59.19	38.34	-	ARS1
AJK-2	+	-	16	10.3	5.9	1.64	ARS2
AJK-3	+	+	16	72.32	62.45	1.78	ARS3 *Stenotrophomonas rhizophila*
AJK-4	+	+	2	1.88	0	2.25	ARS4 *Bacillus* sp.
AJK-5	+	-	2	0	0	1.44	ARS2
AJK-6	+	+	2	0.27	0	7.89	ARS3
AJK-7	+	+	8	7.56	0.12	1.53	ARS2 *Acetobactor pasteurianus*
AJK-8	+	+	19	0.56	0	-	ARS4 *Bacillus* sp.
AJK-9	+	-	4	77.98	67.54	2.78	ARS3 *Stenotrophomonas*

^∗^All isolates produced substantial amount of gum on LB agar plates but non-solubilized Zinc oxide or Zinc sulfate *in vitro*. ARA, Acetylene reduction assay; IAA, Indole acetic acid was tested with and without the addition of precursor; Data are the mean values of three replicates each and all experiments were repeated twice for confirmation of results. The symbol, + representing the positive reaction or the presence of the traits while the symbol, - shows the negative reaction or absence of the component/trait.

Acetylene reduction assay (ARA) showed seven isolates including both rhizosphere and endophytic bacteria have the nitrogenase activity ranging from 1.44 to 7.89 μmol C_2_H_4_ mg protein/h. Putative *Stenotrophomonas* sp. isolate AJK-6 showed maximum ARA.

**FIGURE 1:**
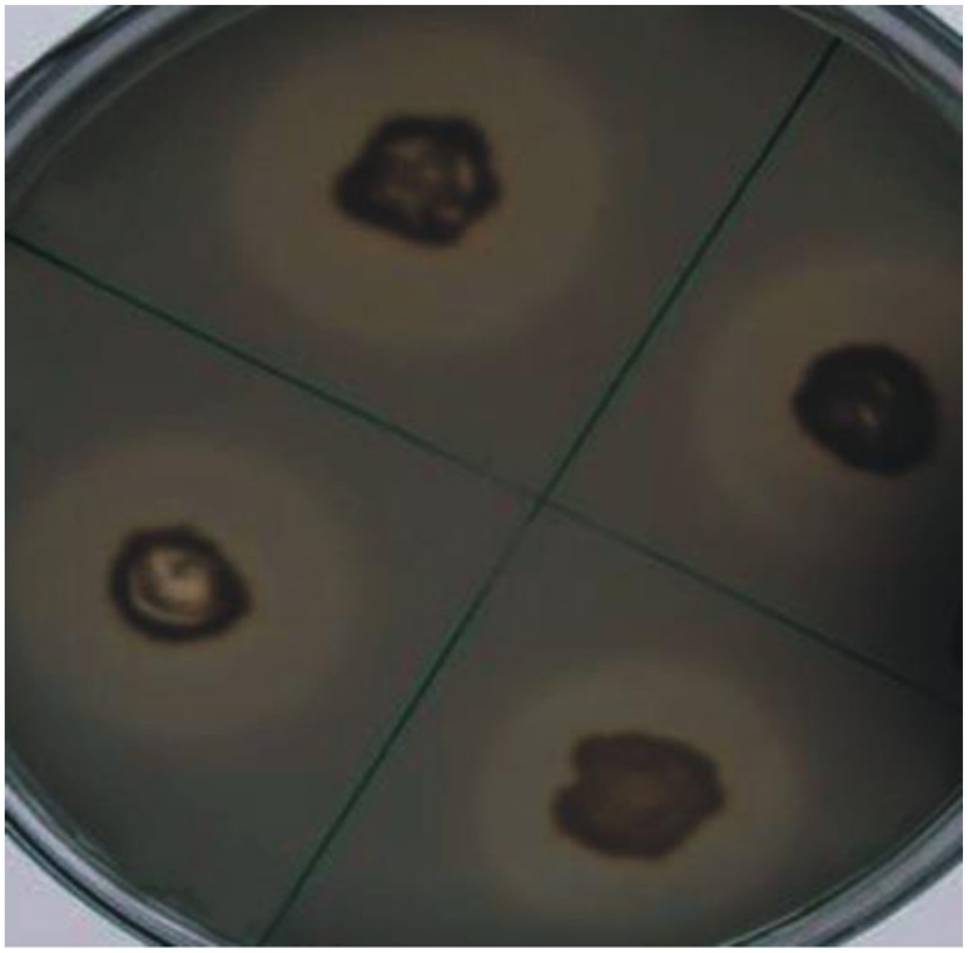
Solubilization of inorganic tri-calcium phosphate *in vitro* by bacterial isolate AJK-8 after 7 days of growth at 28 ± 2^∘^C. The formation of halo zone around the colonies shows the solubilization of the inorganic phosphate.

Four bacteria including three rhizospheric (AJK-1, AJK-2, AJK-3) and one endophytic (AJK-8) were able to form clear zone on Pikovskaia’s agar plates after 7 days of incubation. When quantified spectrophotometrically, maximum P was solubilized by putative *Bacillus* sp. AJK-8 which was an endophytic strain and formed a clear zone of 3 mm diameter on Pikovskaia’s agar plate (**Figure 1**). The range of P-solubilization varied from 2 to 19 μg/mL. We could not found any bacteria able to solubilized zinc oxide or zinc sulfate during this study.

### Root Colonization Potential

**FIGURE 2:**
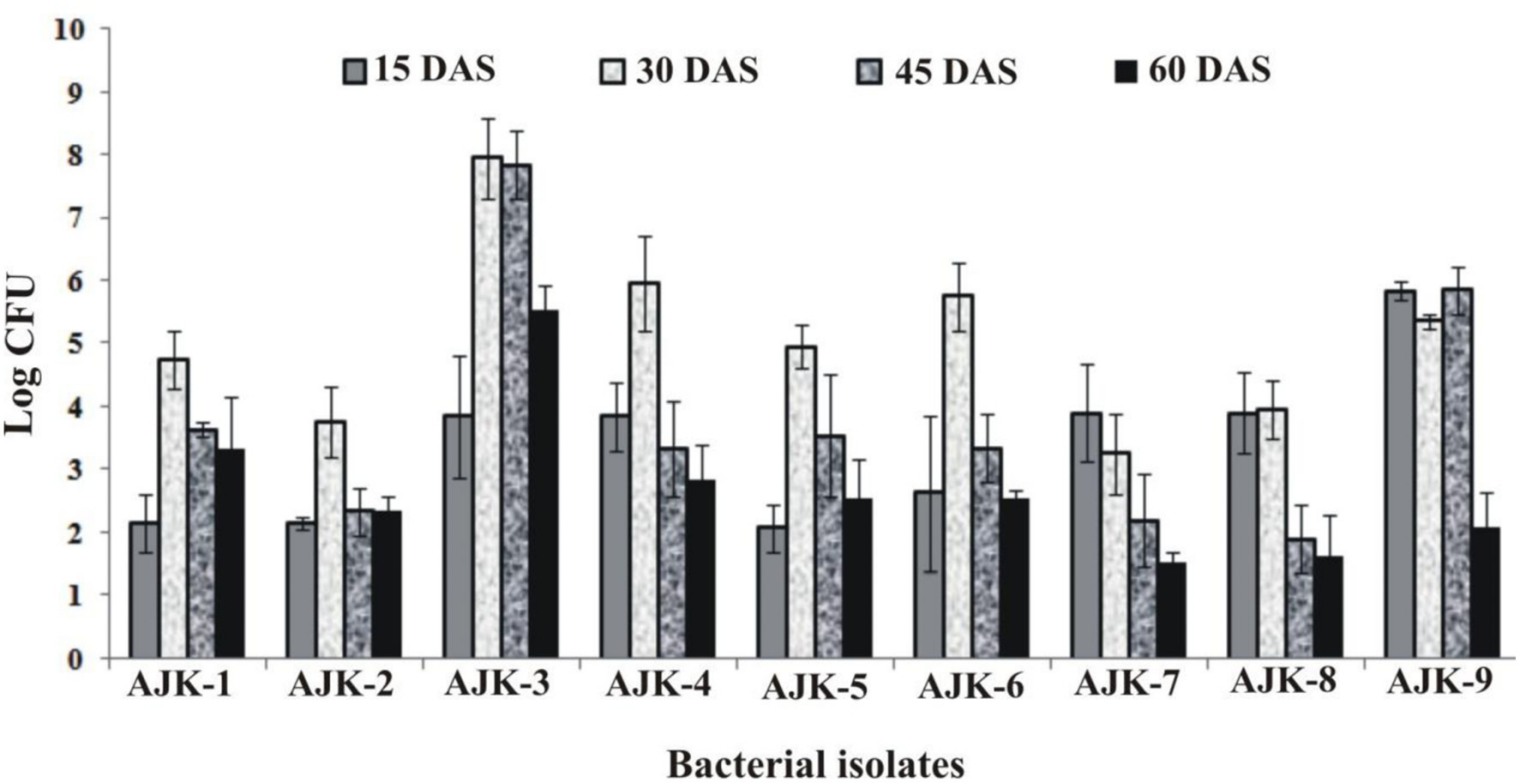
Population density of different bacteria inoculated to wheat at different time intervals under axenic conditions. The error bars represent the least significant difference among treatments at *P*≤ 0.05.

Bacterial population size was determined by plate count method on LB agar at different time intervals. We observed that all rhizospheric as well as endophytic bacteria were able to colonize wheat plant roots and showed persistence in the rhizosphere up to 60 days after inoculation (**Figure 2**). Plants were grown in growth pouches for this experiment; hence, we could not determine the population size at maturity. Maximum colonization was recorded between 30 and 45 days post inoculation. Rhizosphere strain AJK-3 showed maximum number of bacteria at all times as compared to other bacterial isolates.

### Plant Growth Parameters

**FIGURE 3:**
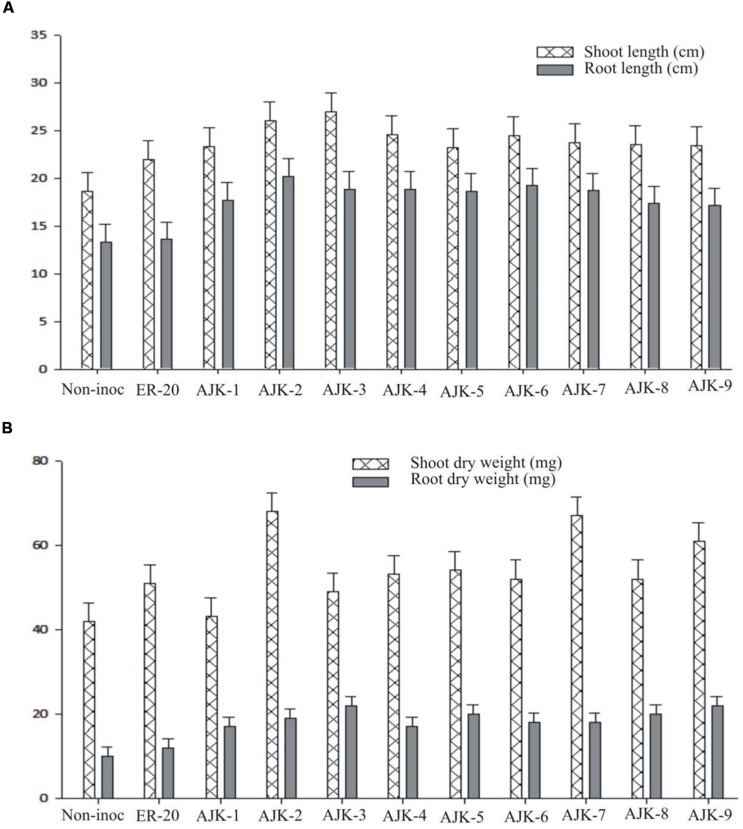
Effect of PGPR inoculation on shoot and root length **(A)** and shoot/root dry weight **(B)** of wheat variety Inqlab grown in growth pouches under axenic conditions. The error bars represent the least significant difference among treatments at *P*≤ 0.05.

**FIGURE 4:**
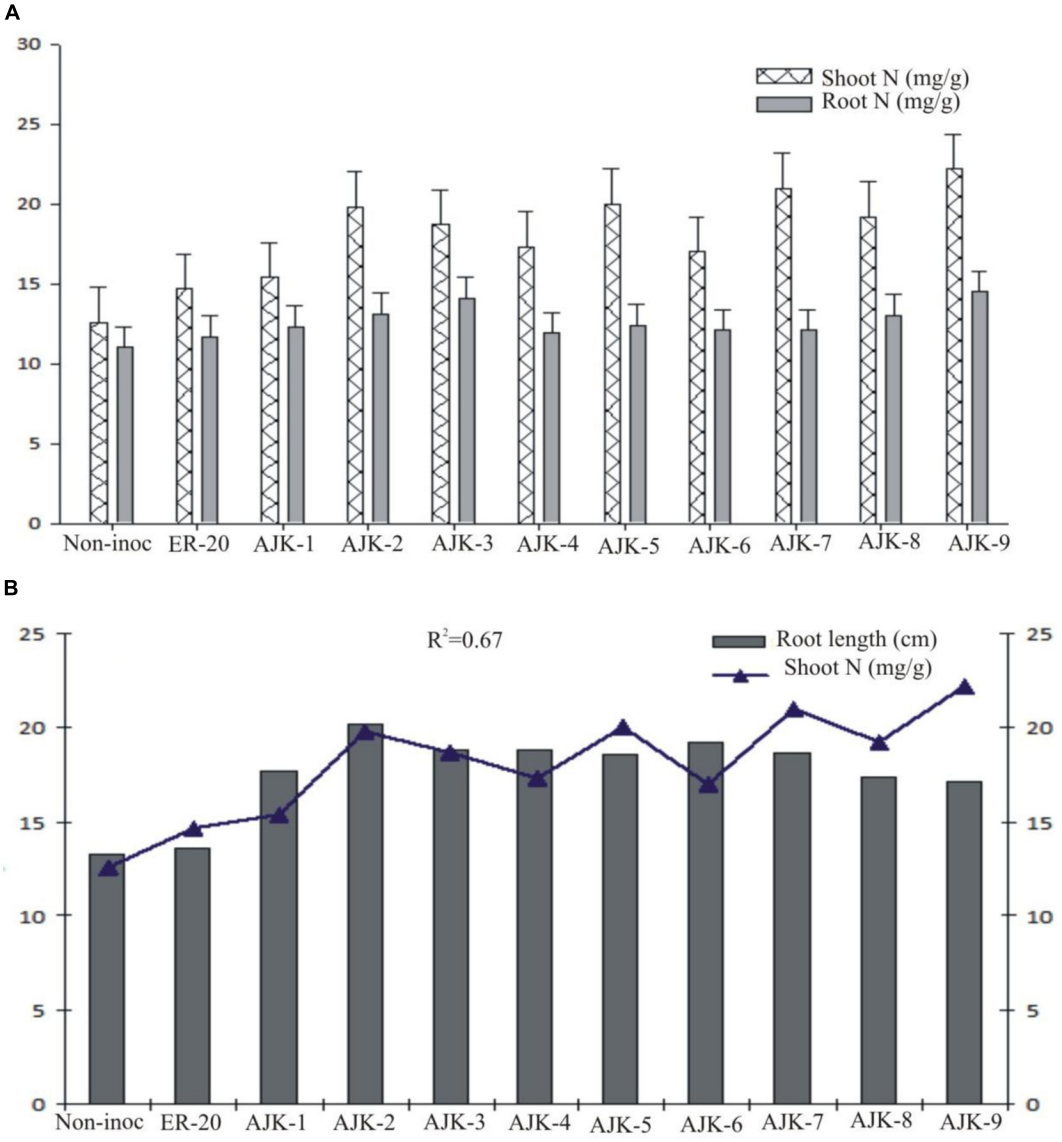
Nitrogen contents (mg g^-1^) in shoots and roots **(A)** and relationship between shoot N contents vs. root length, the error bars represent the least significant difference (LSD) among different treatments at *P*≤ 0.05 **(B)** of PGPR-inoculated wheat variety Inqlab grown in growth pouches under axenic conditions.

The bacterial isolates exerted a significant influence on wheat growth characteristics (**Table 3**; **Figure 3**). Comparisons were made among *Azospirillum* (ER-20) inoculated positive control and a non-inoculated control (with Hoagland N and P). The relative increase in shoot and root length due to bacterial isolates ranged between 25–45% and 29–52%, respectively, over the un-inoculated control whilst the corresponding increase in the shoot and root biomass ranged between 2–62% and 100–172%, respectively (**Figure 3A,B**). Similarly, bacterial isolates significantly increased N content both in shoot and root compared to un-inoculated control and Er-20 (**Figure 4A**). The relative increase varied between 22–76% for shoot and 10–32% for root over the un-inoculated control. The correlation between root length and shoot N contents was positive and significant, i.e., *R*^2^ = 0.67 (**Figure 4B**). The efficacy of different isolates for growth characteristics was variable. Bacterial isolates AJK-2, AJK-9, AJK-3, and AJK-7 performed significantly better than others. Overall, the effect of bacterial inoculation was more pronounced on root than shoot.

**Table 3:** Effect of bacterial isolates on the growth characteristics of wheat grown in pouches under axenic conditions.

Treatments	Shoot length (cm)	Shoot fresh weight (mg plant^-1^)	Shoot dry weight (mg plant^-1^)	Root length (cm)	Root fresh weight (mg plant^-1^)	Root dry weight (mg plant^-1^)
control	22.0	90	42	15.30	25	7
Er-20	18.61	82	51	15.58	18	9
AJK-1	23.3	105	43	19.73	35	14
AJK-2	26.0	129	68	22.15	42	16
AJK-3	27.0	109	49	20.85	36	19
AJK-4	24.57	118	53	20.83	47	14
AJK-5	23.20	111	54	20.63	41	17
AJK-6	24.49	108	52	21.20	38	15
AJK-7	23.69	10	67	20.77	39	15
AJK-8	23.46	117	52	19.36	51	17
AJK-9	23.40	135	61	19.13	49	11
LSD (*P*≤ 0.05)	3.96	6.84	5.30	3.67	2.51	5.34
SEM	1.34	12.0	8.0	1.25	13.0	1.4

*Control, non-inoculated, no bacterial inoculums but plants were provided with full strength Hoagland containing N and P nutrients; Er-20, Positive control strain; SEM, standard error of means; Data are the mean values of 10 replicates; LSD, least significant difference.*

## Discussion

The PGP potential of Rhizobacteria isolated from rhizosphere and endo-rhizosphere of wheat grown in the mountain region (not previously explored) was examined and characterized through polyphasic approach. Based on morphological observations, we found two putative *Bacillus* sp. strains including one rhizospheric isolate AJK-4 and one endophytic isolate AJIK-8. Similarly, we found *Stenotrophomonas* sp. strains both in the rhizosphere (AJK-3) as well as endosphere (AJK-9) of wheat. The 16S rRNA sequence analysis of bacterial strain AJK-3 showed 99% similarity with *S. rhizophila* type strain DSM 14405 which has been reported as a plant associated bacterium showing separate physiological and genetic cluster with low DNA–DNA hybridization value (<50%) from clinical and environmental strains (Wolf et al., 2001).* S. rhizophila* strains are plant associated and have been isolated from the rhizosphere of oilseed rape and from the rhizosphere, and potato tuber. Endophytic colonization of this bacterium has also been reported along with its antagonistic activity against plant pathogenic fungi, e.g., *Verticillium dahliae, Rhizoctonia solani, Sclerotinia sclerotiorum*, and the human pathogenic fungus *Candida albicans*. The endophytic isolate AJK-9 showed 95% similarity with *Stenotrophomonas* sp. and shared many of its beneficial characteristics resembling *Stenotrophomonas* strain AJK-3. We found that both *Bacillus* and *Stenotrophomonas* are colonizers of wheat plant in the rhizosphere as well as root interior in that region. Endophytic strain AJK-7 was identified as *Acetobactor pasteurianus*. *Acetobacter* sp. are obligatory aerobic, nitrogen-fixing bacteria that are known for producing acid as a result of metabolic processes. *Acetobacter diazotrophicus* is also a plant endophyte and has been said to be capable of excreting about half of its fixed nitrogen in a form that plants can use. The study indicated that the biochemical tests of bacterial identification and characterization can only be used to some extent to discriminate among the bacterial strains, but could not distinguish among the closely related ones. While, full-length 16S rRNA sequencing provides information regarding separate entity of each isolate from the wheat rhizosphere. Previous studies indicated I6S rRNA sequence analysis as an authenticated technique used to study bacterial isolates at species level (Imran et al., 2010; Alam et al., 2011).

*In vitro* screening for characteristics commonly associated with plant growth promotion revealed that seven bacteria were able to produce IAA in a range of 0.27–77.98 μg/mL, indicating a substantial variability among rhizosphere and endophytic wheat isolates for IAA production. The potential of bacterial isolates to produce IAA indicates their ability to use as growth hormones or growth regulators. Our results were in agreement with the previous study where the PGPR from the rhizosphere of *Brassica campestris* had shown to produce 6.02–29.75 μg/ml of IAA (Poonguzhali et al., 2008). The variation in the ability of PGPR to produce IAA found in the present study had also been reported earlier (Mansour et al., 1994; Zahir et al., 2000). This variation is attributed to the various biosynthetic pathways, location of the genes involved, regulatory sequences, and the presence of enzymes to convert active free IAA into conjugated forms (Patten and Glick, 1996). The production of IAA by bacteria isolated from rhizosphere of different crops, i.e., peanut, maize, wheat, and rice had already been reported in number of studies (Dey et al., 2004; Cakmakci et al., 2007; Mehnaz et al., 2010). The amount of IAA detected in the present study (with and without tryptophan) seems relatively higher than reported earlier, indicating that soils under investigation have bacteria that have the characteristics most commonly sought for use in growth enhancement of plants.

The beneficial effect of PGPR in maintaining adequate levels of mineral nutrients especially the P in crop production had been previously reported (Rodriguez and Fraga, 1999; Saravanan et al., 2007). In our study, four bacterial isolates were found efficient solubilizer of phosphate. The ability of PGPR strains to solubilize insoluble P and convert it to plant available form is an important characteristic under conditions where P is a limiting factor for crop production. In two different studies, very limited number of P-solubilizers (23.5% of the total tested strains and five out of the 207 isolates) has been reported (Hameeda et al., 2006; Islam et al., 2010). The soil phosphate solubilizing strains can increase the availability of phosphorus to plant by mineralizing organic phosphorus compounds and by converting inorganic phosphorus into more available form (Bar-Yosef et al., 1999). Phosphate solubilization is mainly due to the production of microbial metabolites including organic acids which decreases the pH of the culture media (Puente et al., 2004; Sahin et al., 2004; Shahid et al., 2012). The presence of P-solubilizing microbial population in soils may be considered a positive indicator of utilizing the microbes as biofertilizers for crop production and beneficial for sustainable agriculture. The results of nitrogen fixation by ARA method indicated that fairly large population of wheat associated nitrogen fixers are present in the soils and can be beneficial to improve nitrogen nutrition of wheat and other crops.

All the bacterial isolates significantly increased shoot and root length, shoot and root dry weight, and also enhanced the N contents of inoculated wheat seedlings. The plant growth promotion could be the result of the beneficial functions of applied PGPR isolates, like plant growth hormone production, nitrogen fixation, and P solubilization. As the inoculated plants were not supplied with any additional source of N or any form of soluble P, the higher amount of N detected in the shoot or roots of inoculated plants as well as growth promotion may be attributed to the bacterial-assisted growth enhancement phenomenon. In addition to some other parameters positively influenced the growth of plant, auxin production by the isolates is proposed as a major means of attaining growth promotion (Deepa et al., 2010). Furthermore, the inoculation of PGPR having multi-functional traits is better than having single traits (Imran et al., 2014). IAA is involved in root initiation, cell division, cell enlargement, increases root surface area, and consequent access to soil nutrients by enhanced formation of roots (Dey et al., 2004; Gray and Smith, 2005). Auxin production has been proposed as a major means of attaining early growth promotion in wheat (Khalid et al., 2004) along-with P-solubilization (Rajput et al., 2013). The response of plants to different isolates was variable which may be attributed to their individual traits and rhizospheric competencies. Most of the bacteria showed good survival and persistence in the rhizosphere. The significant increase in growth and N level both in shoot and root upon isolates application is a clear indicative of the fact that the bacterial isolates have been able to provide better nutrient flux to the plant host which resulted in the increase of the plant biomass and N accumulation. The increase in root length due to the applied isolates may also contributed to increase N uptake in plant shoot as both the parameters were significantly correlated in the study (*R*^2^ = 0.67).

## Conclusion

This is a basic study that has provided an insight into the bacterial community present in the mountainous region of Rawalakot, AJK, Pakistan. We have demonstrated efficient N_2_-fixing, P-solubilizing, and IAA-producing bacteria present among the natural population. These characteristics are considered as important PGP traits and have been found effective in positively improving the growth and N contents of tested wheat plants. These isolates offer potential in field applications as PGP agents in wheat. Further studies should be focused on the detailed molecular and functional characterization of these PGPR for practical applications in the field.

## Conflict of Interest Statement

The authors declare that the research was conducted in the absence of any commercial or financial relationships that could be construed as a potential conflict of interest.
